# Insight-related beliefs and controllability appraisals contribute little to hallucinated voices: a transdiagnostic network analysis study

**DOI:** 10.1007/s00406-020-01166-3

**Published:** 2020-07-14

**Authors:** Elisavet Pappa, Emmanuelle Peters, Vaughan Bell

**Affiliations:** 1 Department of Psychiatry, University College London, London, UK; 2 Department of Psychology, Institute of Psychiatry, Psychology and Neuroscience, King’s College London, London, UK; 3 Psychological Interventions Clinic for Outpatients with Psychosis (PICuP), South London and Maudsley NHS Foundation Trust, London, UK; 4 Research Department of Clinical, Educational and Health Psychology, UCL Centre for Clinical Psychology, University College London, Gower Street, London, WC1E 6BT UK

**Keywords:** Psychosis, Network analysis, Predictability, Metacognition, Auditory hallucinations

## Abstract

Hallucinated voices cause high levels of distress and disability. Current theories suggest that insight-related beliefs, about internal or external origin, perceived source location, and appraisals of controllability are important in mediating the impact of these experiences but previous findings have been mixed. We report two open code and open data network analytic studies of items in the Psychotic Symptoms Ratings Scale for auditory verbal hallucinations (PSYRATS-AH) in a large sample of patients with hallucinated voices to examine the network structure of items at (1) first assessment, and (2) differences over two consecutive assessments during a wait-list period. Networks were generated using least absolute shrinkage and selection operator (LASSO) and extended Bayesian information criterion (EBIC) with node predictability. In Study 1 (*N* = 386), we report that insight-related items made a negligible contribution to hallucinated voices and the controllability appraisal made at most a modest contribution. Items relating to distress and negative content were the most central and most predicted by the wider network. In Study 2 (*N* = 204), we tested the longitudinal stability of the structure of hallucinated voices over a period of several months, finding a small change in total hallucination score and global strength but no clear evidence for an alteration in the structural relationship. The insight-related and controllability items remained as least influential over time. Insight-related beliefs and controllability appraisals may contribute less than previously thought to distressing hallucinated voices although we do not discount that other appraisals may remain important.

**Electronic supplementary material:**

The online version of this article (10.1007/s00406-020-01166-3) contains supplementary material, which is available to authorized users.

## Background

Auditory verbal hallucinations are present in up to 70% of patients with a diagnosis of schizophrenia, almost a quarter of patients with bipolar disorder, and contribute to both distress and disability in affected people [1]. However, the differing contributions of appraisals, perceptual and affective components are still poorly understood and a better understanding of the interrelationships between these components has been highlighted as a research priority [2].

The relationship between appraisals and other characteristics of hallucinated voices has been of interest due to their potential role in mediating impact. Traditionally, one of the most important appraisals relates to the perceived origin of voices, with ‘insight’ ascribed to the voice hearer if they believe the voices are generated within the self and ‘lack of insight’ ascribed to the voice hearer if they believe the voices originate from external sources [3]. The perceived auditory ‘location’ of the voices has also been considered to be a marker of clinical significance, with those perceived as coming from outside the head categorised as ‘true hallucinations’ and those perceived to be coming from inside the head designated as ‘pseudohallucinations’ [4]. Notably, some non-diagnostic cognitive models also stipulate internal versus external attributions as importing in determining whether the experience becomes a psychotic symptom [5]. The importance of location appraisals for judging hallucination severity has received mixed support. Stephane et al. [6] found that patients with schizophrenia who heard hallucinations inside their head were more likely to demonstrate memory source monitoring deficits, while Docherty et al. [7] found that internally located hallucinations were more intrusive and distressing. However, no reliable associations were found by Copolov et al. [4] in large sample of patients with psychotic disorders, neither were they found by Oulis et al.’s [8] study on patients admitted with acute psychosis, and they were only associated with number of hallucinated words and utterances in a study by Nayani and David [9]. Despite these mixed results, these characteristics are still cited in contemporary psychiatric textbooks as unequivocal markers of clinical severity [10, 11].

Morrison et al. [12] have cited metacognitive beliefs relating to the controllability of thoughts as a key component in hallucination proneness. A recent meta-analysis [13] examined judgements about the controllability of thoughts, the controllability of the person’s own mind, as well as confidence in the accuracy of the person’s own mind, and found only a moderate association between these factors and distress in clinical samples—which was reduced to a weak association when co-morbid symptoms were controlled for. However, appraisals regarding perceived controllability of voices themselves, rather than thoughts in general, have been found to characterise clinical as opposed to non-clinical voice hearers [14] (i.e. people not distressed or impaired by their voices), patients with first episode psychosis [1] and patients with more severe voice hearing symptoms [7].

Nevertheless, one drawback with existing studies in this area is that they have tended to rely on composite measures of severity that sum a range of characteristics like distress, intrusiveness and insight into a single metric—potentially obscuring structural relationships and interactions between insight-related beliefs, appraisals, and the affective and perceptual components of voices. Similarly, many studies are cross-sectional in nature meaning it is not possible to see how the relationship between different characteristics changes over time—something that requires longitudinal analysis.

One way of more effectively addressing the interaction of components in psychopathology is through the use of network analysis which estimates plausible candidates for causal interactions based on statistical relationships between symptom component measures, controlled for every other variable in the network [15]. One additional advantage of this method is that the potential importance of each element for the overall function and coherence of the network can be estimated using graph theoretic metrics [16]. Such networks therefore provide estimates of the significance of each characteristic in the overall network and potentially suggest beneficial points of intervention. However, to date, only a handful of studies have applied this method to understanding psychosis. For example, Isvoranu et al. [17] examined the inter-relationships between trauma and psychotic symptoms, Bell and O’Driscoll [18] and Murphy et al. [19] examined paranoia and the wider psychosis phenotype respectively in the general population, and van Rooijen et al. [20] examined psychosis-related symptoms in patients with psychosis. None to date have directly investigated hallucinated voices.

Consequently, we applied network analysis to examine the contribution of appraisals about origin, perceived source location, and controllability, to the structure and temporal stability of auditory hallucinations networks in a large sample of patients with psychosis using items from the Psychotic Symptom Rating Scales for Voices (PSYRATS-AH; [21]). We report two studies. For the first, we examined the relative importance of each of these components in a large sample of patients with hallucinated voices who attended an initial assessment at a clinic for psychological interventions for psychosis. For the second, following Santos et al. [22] we tested the longitudinal stability of the network in a sub-sample of patients who were followed-up over time while they were on a waiting list for intervention, allowing us to examine how these factors alter over time.

## Methods

### Setting

Data were collected from clinical assessments from 2003 to 2018 at the Psychological Interventions Clinic for outpatients with Psychosis (PICuP) based at the Maudsley Hospital in the South London and Maudsley National Health Service Foundation Trust. PICuP accepts referrals mainly from four boroughs in South London. Referral criteria for PICuP is “a diagnosis or suspected diagnosis of psychosis (including bipolar disorder), or presence of psychotic symptoms (for instance as a result of trauma)”. Referrals are screened and those with confirmed psychotic symptoms are invited for an assessment. They are a highly diverse group in terms of social, economic, forensic, health and mental health (previously described in Peters et al. [23]).

### Sample and procedures

For Study 1, we included patients who reported auditory hallucinations at the point of initial assessment (indicated by a positive PSYRATS-AH score) and had no missing data on any scale item, which left a total of 386 patients included in the analysis.

For the longitudinal analysis in Study 2, we included patients who had a positive PSYRATS-AH score at initial assessment and complete PSYRATS-AH assessments at both initial and second assessments, which left 204 patients included in the analysis. These two assessments are the initial assessments that form part of several standard clinical assessments that occur during the process of therapy. The first assessment occurs when patients are initially referred to the service and are added to the waiting list. The second, pre-therapy assessment occurs just prior to being allocated a therapist for the start of treatment. While on the waiting list the patient is living in the community, either under the care of a community mental health team or a general practitioner.

Assessments are conducted by assistant psychologists trained in conducting the evaluations that includes the PSYRATS-AH and several other measures with the full assessment lasting approximately 45–90 min. Demographic data are collected through the standard ‘Patient Registration Form’. Ethical approval for the use of these data in research was granted by the London-Dulwich Research Ethics Committee (ref: 15/LO/1831) and only patients who consent to their anonymised data being used in research are included in the database. Therefore, the study has been conducted in accordance with the ethical standards laid down in the 1964 Declaration of Helsinki and its later amendments.

### Measures

Auditory hallucinations were measured with the Psychotic Symptom Rating Scale for Voices (PSYRATS-AH; [21]) a widely used and clinically validated measure of auditory verbal hallucinations (internal consistency; *α* = 0.75) that takes the form of a semi-structured, 11-item, clinician-rated interview [24]. Each item is rated on a five-point ordinal scale from 0 (absent) to 4 (severe) with overall score ranging from 0–44 and includes items measuring frequency, disruption of life, duration, loudness, amount and degree of negative content (two items), amount and intensity of distress (two items), location (i.e., whether the voices sound like they are coming from inside or outside the head), belief about origin of the voices (i.e., whether they are internally or externally generated), and perceived controllability over the voices (i.e. can they bring them on or dismiss them). Each item on the PSYRATS-AH was included as a separate node in the network analysis.

### Statistical analysis and data availability

The statistical programming language *R* (version 3.5.3) was used to conduct all analyses. Open data and code have been made available that allow all main analyses to be reproduced. However, we have not included some demographic and descriptive clinical variables in the open data set to insure against re-identification. All analyses were completed on a 64-bit × 86 Linux platform. The analysis code and non-identifiable data for reproducing the analyses is available online at: https://osf.io/qkn6z/

For the first analysis, we used the *mgm* package (version 1.2.5; [25]) to generate mixed graphical models to estimate the network using Least Absolute Shrinkage and Selection Operator (LASSO; [26]) using Extended Bayesian Information Criterion (EBIC) to select the best model [27]. This method produces an estimated network structure that maximises the probability of deriving the genuine structure in the population. In addition, we also calculated predictability for each node in each network [28, 29]. Predictability indicates how the value of each node is predicted by connected nodes potentially giving a more plausible metric of practical importance in terms of targets for interventions [29]. High predictability indicates that variance in a node is determined by mutual interactions between connected nodes. In summary, centrality metrics attempt to estimate how much the node affects the network, whereas predictability metrics attempt to estimate how much the network affects the node.

In the network visualisations, strengths of associations are represented by the thickness of the lines between nodes with positive relationships in green and negative relationships in red. The extent of node predictability is visualised by a ring (equivalent to a ‘donut graph’) around the edge of each node. Network layouts were generated using the *qgraph* (version 1.6.1; [30]) using the Fruchterman–Reingold algorithm [31].

We subsequently estimated the following centrality metrics for each node [32] using *qgraph*: betweenness, referring to the number of times that a node lies in the shortest path between two other nodes in the network; closeness, defined as the average distance from a node to all other nodes in the network; strength, as the sum of weighted correlation coefficients of all the edges connected to a node; and expected influence, a measure of centrality that adjusts for the combined influence of positive and negative associations [33].

For Study 2, we compared total PSYRATS-AH scores between assessments using a paired t-test and Pearson’s *r*. We then generated networks for the first and second assessments using the same statistical approach as described in Study 1. Following Santos et al. [22] these networks were subsequently compared using *NetworkComparisonTest* package for *R* (NCT; [34]) modified for ordinal data [35]. The NCT is a two-tailed permutation test that uses random regrouping of participants from the networks and calculates the differences between the networks [34, 36]. We tested for statistical difference in: (1) invariant network structure—whether the overall network structure is significantly different between time points; (2) edge strength, testing whether edges are different across networks; and (3) global strength—whether the overall level of connectivity is different. We also estimated centrality metrics and predictability values for each node in the two networks.

Finally, we assessed the stability of centrality metrics using an *m out of n* bootstrap method using the *bootnet* package [37] to estimate edge-weight reliability and a bootstrap analyses to estimate edge-weight reliability for *mgm* networks using the technique reported in Fried et al. [38].

## Results

### Study 1: Network structure of auditory verbal hallucinations

#### Descriptive statistics

Of the 386 included patients, 210 (55.4%) were male, 169 (44.6%) were female, with seven patients recorded as having missing data (which may include ‘prefer not to say’). The mean age of patients was 40.84 (SD = 10.2). Ethnicity for the sample was recorded as: White British (*N* = 133), White English (*N* = 6), Irish (*N* = 11), White Other (*N* = 20), Black Caribbean (*N* = 19), Black British (*N* = 48), Black African (*N* = 37), Other African (*N* = 12), Indian (*N* = 6), Pakistani (*N* = 1), Bangladeshi (*N* = 3), Other (*N* = 57), and with 33 patients recorded as having missing data for ethnicity.

Mean PSYRATS-AH total score was 27.57 (SD = 6.42; range 2–43). Primary diagnoses were only recorded in the research database of the clinic from 2013 meaning diagnoses are not available for all patients. From available data, primary diagnoses were recorded as Schizophrenia (*N* = 84), Depressive/mood disorder (*N* = 27), anxiety disorder (*N* = 10), bipolar disorder (*N* = 6), PTSD (*N* = 5) and other neurotic disorder (*N* = 1). Secondary and subsequent diagnoses were not available. Antipsychotic prescribing was not recorded in the research database, however, a previously conducted clinical trial of psychological therapy with participants referred to the same service reported an antipsychotic medication prevalence of 96% [39].

#### Network structure

**Fig. 1:**
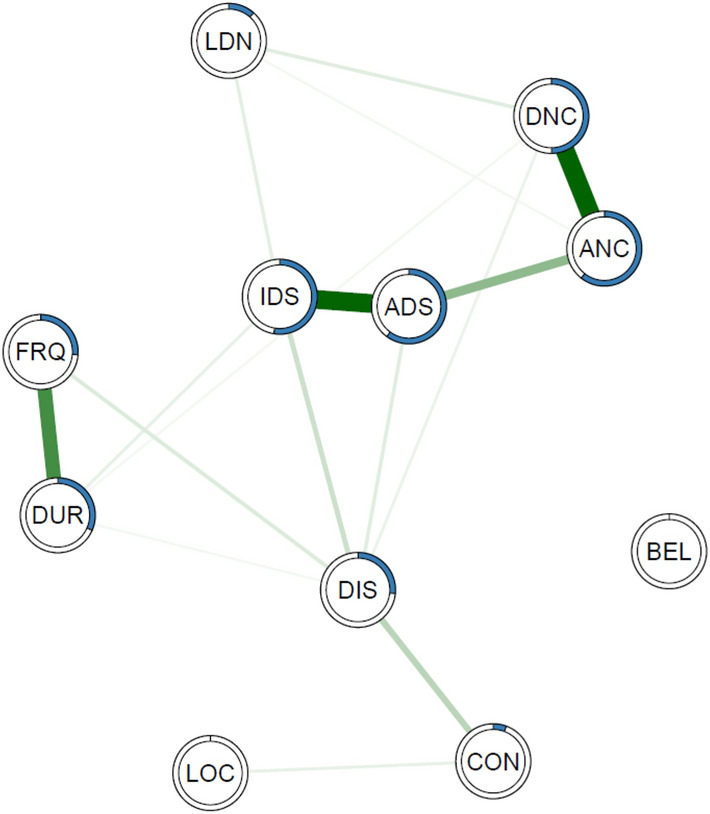
MGM predictability networks of PSYRATS-AH items of auditory hallucinations. The thickness of the line indicates the strength of the relationship between nodes. Blue ring around each node represents the proportion of the variance of this item explained by all other items in the network*.* PSYRATS-AH items: *CON* controllability of voices, *LOC* location, *BEL* belief re: origin of voices, *DUR* duration, *DIS* disruption to life caused by voices, *LDN* loudness, *FRQ* frequency, *IDS* intensity of distress, *ADS* amount of distress, *ANC* amount of negative content, *DNC* degree of negative content

**Fig. 2:**
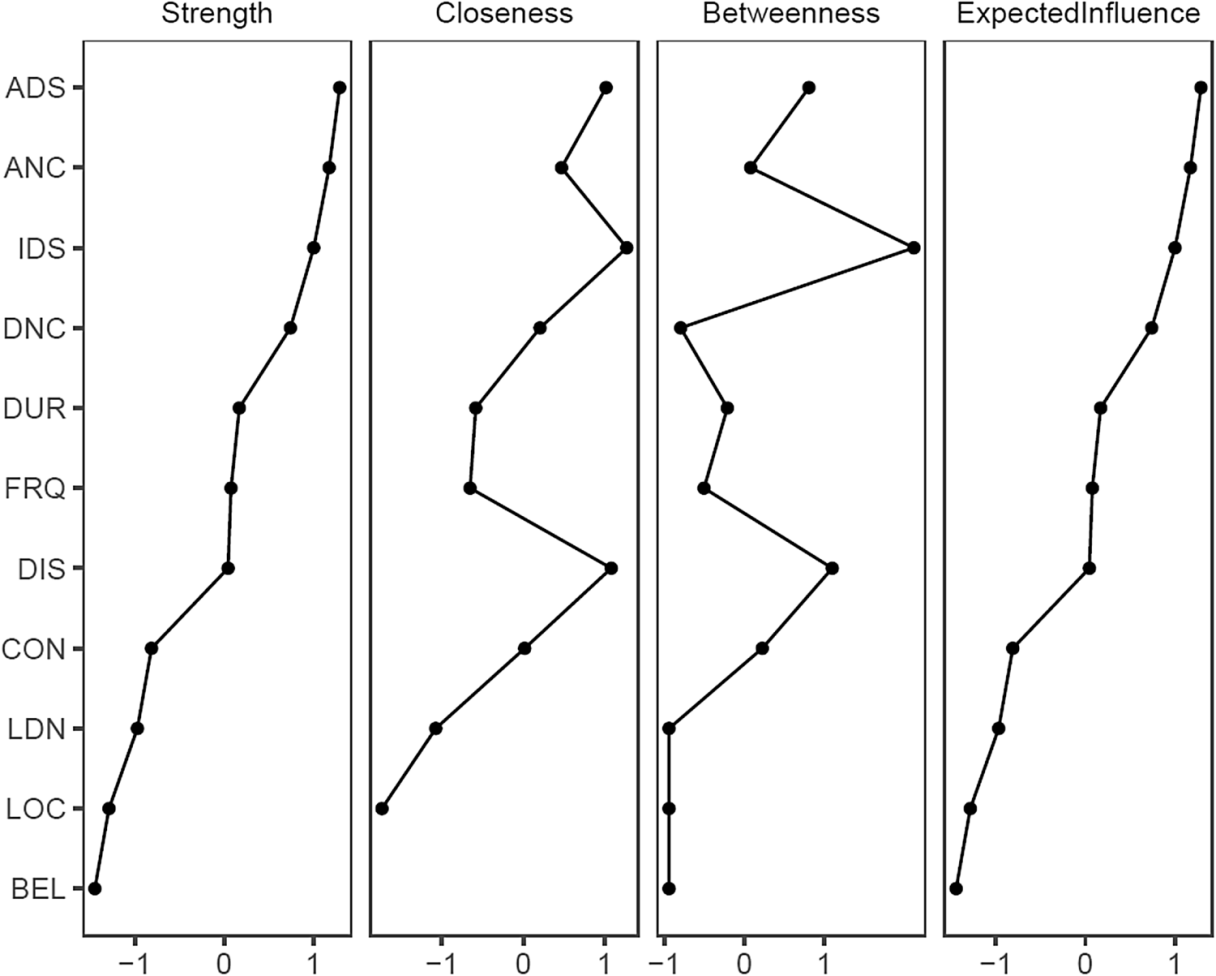
Centrality metrics for PSYRATS-AH items in network of auditory hallucinations, ordered by Expected Influence

The network is displayed in Fig. 1. All nodes form part of the network except the item ‘belief regarding the origin of voices’ (BEL) which is isolated due to having estimated edges of value zero with other nodes. Although Location (LOC) does form part of the overall network it is connected by a single edge of low edge weight and has an *R*^2^ predictability metric of 0. These items score lowest for overall expected influence (BEL = − 1.45, LOC = − 1.29). The additional appraisal item Controllability (CON) has a relatively low expected influence (− 0.81; 8th out of 11 nodes) and predictability (*R*^2^ predictability of 5.7%) although it does contribute to overall network structure. Notably, it is most strongly connected to disruption to life (DIS) suggesting a mutual interaction between these nodes. Numeric predictability scores are reported in Table S1 of the supplementary material (Fig. 2).

The most central, influential, and most highly predicted by variation in other network nodes are typically those nodes related to distress. Intensity of distress (IDS), amount of distress (ADS) and amount of negative content (ANC) are estimated to be the most influential nodes in the network as well as those with values that are most predicted by the values of the other nodes in the network. Notably, disruption of life (DIS) has the second highest value in terms of closeness (1.07) and betweenness (1.10), following IDS, which indicates it has the second largest average distance from all other nodes in the network.

#### Reliability of network estimates

The results of bootstrapped values for edge-weight reliability are included in the Supplementary Materials.

### Study 2: Temporal stability of network structure for hallucinated voices

### Descriptive statistics

Of the 204 included patients, 104 (51.2%) were male, 99 (48.8%) were female and one person had missing data. The mean age of patients was 40.95 (SD = 9.74). Ethnicity for the sample was recorded as: White British (*N* = 67), White English (*N* = 5), Irish (*N* = 7), White Other (*N* = 11), Black Caribbean (*N* = 10), Black British (*N* = 30), Black African (*N* = 21), Other African (*N* = 7), Indian (*N* = 3), Pakistani (*N* = 1), Bangladeshi (*N* = 3), Other (*N* = 31) with eight patients recorded as missing data.

As previously, diagnoses are only available for a subset of patients. Primary diagnoses for this sample was recorded as: Schizophrenia (*N* = 39), depressive/mood disorder (*N* = 20), anxiety disorder (*N* = 6), bipolar disorder (*N* = 5), PTSD (N = 1) and other neurotic disorder (*N* = 1).

The mean time period between the two assessments was 117.2 days (SD = 68.4; range 23–524).

An independent samples *t* test indicated that the patients who did not have data for a second assessment and so were excluded from Study 2, were not significantly different in their total PSYRATS-AH scores from patients who were included (*t* = 0.397, *p* = 0.691, Cohen’s *d* = 0.041).

#### Change in scale scores

Mean total PSYRATS-AH score at assessment one was 27.69 (SD = 5.92), at assessment two it was 25.92 (SD = 8.36). Total PSYRATS-AH scores from the first and second assessment correlated (*r* = 0.59, *p* < 0.001) although when tested with a paired samples *t* test the difference between scores was significant (*t* = 3.72; *p* = 0.0003) with an effect size calculated with a Cohen’s *d* of 0.24 indicating a statistically significant decline of small effect between the two assessments.

#### Network structure

**Fig. 3:**
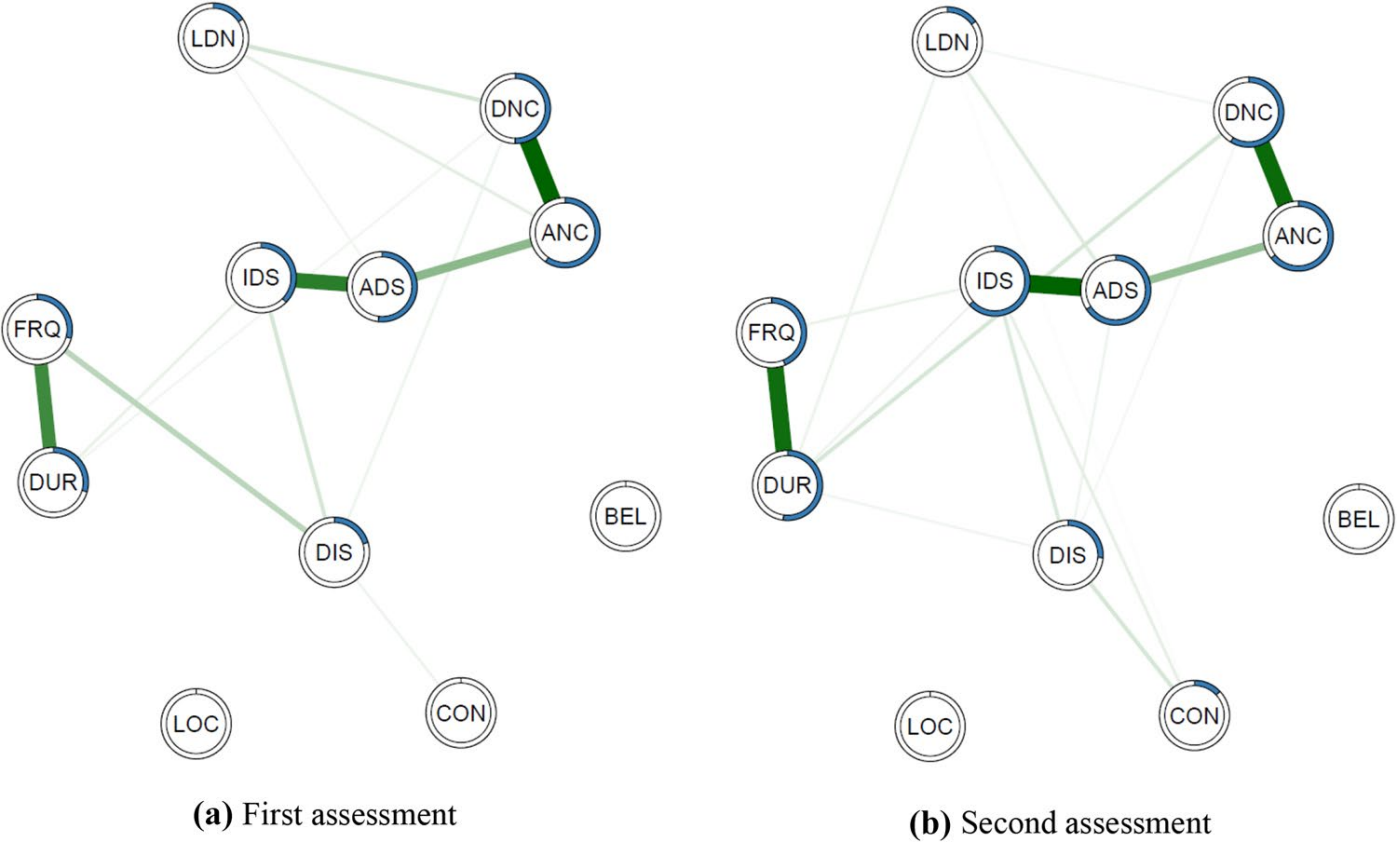
Estimate networks of PSYRATS-AH items on **a** first and **b** second assessment of auditory hallucinations over a period of non-intervention. The thickness of the line indicates the strength of the relationship between nodes. Blue ring around each node represents the proportion of the variance of this item explained by all other items in the network*.* PSYRATS-AH items: *CON* controllability of voices, *LOC* location, *BEL* belief about the origin of voices, *DUR* duration, *DIS* disruption to life caused by voices, *LDN* loudness, *FRQ* frequency, *IDS* intensity of distress, *ADS* amount of distress, *ANC* amount of negative content, *DNC* degree of negative content

Networks with *R*^2^ predictability for each PSYRATS-AH item at assessments one and two are displayed in Fig. 3. The *R*^2^ predictability values ordered by extent of difference are displayed in Table 1.

**Table 1:** *R*^2^ predictability for items at assessments one and two ordered by difference

psyrats-ah item	1st assessment *R*^2^ predictability (%)	2nd assessment *R*^2^ predictability (%)	Difference (%)
Intensity of distress (IDS)	37.4	63.1	25.7
Duration (DUR)	29.4	52.1	22.7
Frequency (FRQ)	29.1	43.6	14.5
Amount of distress (ADS)	51.8	65.8	14.0
Controllability (CON)	0.0	13.0	13.0
Degree of negative content (DNC)	50.1	58.7	8.6
Disruption to life (DIS)	20.5	26.2	5.7
Amount of negative content (ANC)	59.4	64.2	4.8
Loudness (LDN)	15.8	15.0	0.8
Belief about the origin of voices (BEL)	0.0	0.0	0.0
Location (LOC)	0.0	0.0	0.0

#### Centrality metrics

**Fig. 4:**
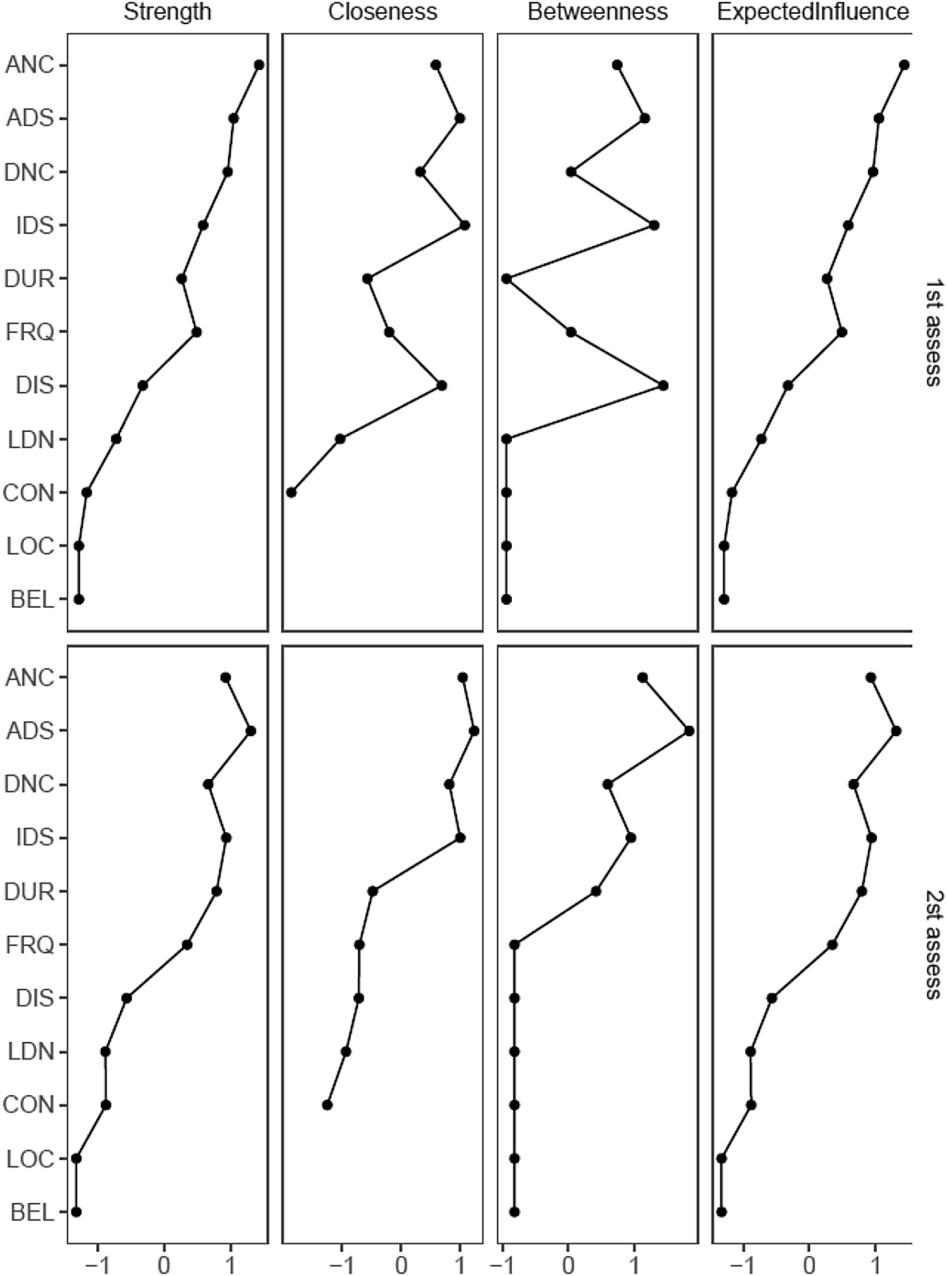
Centrality metrics estimated for auditory hallucinations networks at assessment one and assessment two. Centrality indices are shown as standardized *Z* scores to facilitate comparisons between different networks

Centrality metrics for both networks are displayed in Fig. 4. Centrality metrics tended to be similar across the two assessments, almost identical for strength and estimated influence estimates. Betweenness centrality was most dissimilar between time points although this metric is most sensitive to minor changes in network structure [40]. Loudness (LDN) and ‘Belief about origin’ (BEL) have incomplete closeness metrics due to being isolated nodes in the networks.

#### Differences in global connectivity over time

The results of the *NetworkComparisonTest* between assessments one and two showed the maximum difference between any edge weight was 0.27 (*p* = 0.045). However, we note the p value is only marginally below the traditional cut-off of *p* < 0.05. Post-hoc tests showed 7 out of 121 edges (5.77%) had significantly different weights between the two networks with uncorrected *p* values of less than 0.05. These were: belief about the origin of voices—controllability, belief about the origin of voices—location, belief about the origin of voices—disruption to life, belief about the origin of voices—frequency, frequency—duration, frequency—intensity of distress, intensity of distress—controllability. However, none survived Holm–Bonferroni correction for multiple comparisons.

The two networks showed a difference in global strength values with a global strength value of 2.95 for the first assessment network and 5.10 for the second assessment network (*p* = 0.0025), indicating that the overall connectedness of the second assessment network is increased by 2.15 points compared to the first assessment network. Given the marginal significance of the results of the network structure comparison and lack of difference for specific edge weight difference after correction we conclude conservatively that it is unlikely we can confidently reject the null hypothesis that the networks do not differ. The change in global strength is more convincing and likely reflects the change in PSYRATS-AH total score.

#### Reliability of network estimates

The results of bootstrapped values for edge-weight reliability for both graphs are included in the Supplementary Materials. Given the smaller sample size, as expected, the edge-weight reliability estimates for Study 2 show wider ranges and therefore likely lower reliability than those reported in Study 1.

## Discussion

In this study, insight and controllability appraisal items made relatively small contributions to the overall network structure and to the most clinically significant components with the most central and predictable components tending to reflect distress. The only component that was less influential and predictable than these items was the perceived location of the voice (inside or outside the head), which seemed to be irrelevant to the overall network. In terms of temporal stability, the overall severity score comparison showed a modest statistically significant decrease over the period between assessments and there was evidence of a small change in global strength. However, there was no convincing evidence for an alteration in structure and the appraisal items remained as the least influential.

The fact that insight-related beliefs measured by the PSYRATS-AH (perceived location and belief about origin) contributed almost nothing to the overall network structure either in terms of centrality of predictability raises additional questions about the clinical utility of these criteria in distinguishing ‘clinically significant’ or ‘pathological’ voices [4, 41, 42]. In terms of ‘controllability’ of voices, this item had a modest but detectable potential influence on the network and was most connected to disruption caused by the voices in everyday life. Existing studies on perceived controllability of voices suggests that it is higher in non-clinical voice hearers [14, 43] and in patients with pleasurable voices [44]. Nevertheless, it is not possible to conclude with confidence from our data whether the relationship reflects reverse causality from disruption rather than a measure of metacognitive control. One potential indicator may be that controllability remained low in centrality across the two time points but increased in predictability, potentially suggesting that it is more likely to be affected by, rather than affecting, the network structure of hallucinated voices.

We also note the consistently high centrality and predictability of items relating to distress and negative content. It is also worth noting here that distress-related items remained high in centrality across the two time periods in the longitudinal study, but in terms of predictability, the Intensity of Distress item showed the most change over time out of all the items with other distress and negative content items showing more modest changeability. This may suggest that reducing distress and negative content might be effective in reducing the network of hallucinated voice components as a whole, although given the limited evidence for potential role of controllability and belief about origin, which are common targets for psychological therapy aimed at reducing distress [45], alternative targets may be needed. Considering that the items measuring the frequency and duration of voices seem to show the highest levels of predictability and centrality after the distress items, these may be suitable candidates, although the extent to which they drive distress rather than reflect distress-causing processes remain unclear [46].

Importantly, despite the small to absent contribution of appraisals to the overall experience of hallucinated voices, this does not discount the possibility that other appraisals, evaluations or metacognitive beliefs and processes (reality monitoring, executive control and so on) still play a more central role. For instance, the PSYRATS-AH does not include key appraisals about voices such as omnipotence, identity and social-rank beliefs that are hypothesised to be central to the experience of hearing voices and drive distress and disability [47]. Indeed, a cognitive behavioural therapy intervention focusing on beliefs regarding the ‘power’ and ‘malevolence’ of voices has been successful in reducing compliance with command hallucinations [48, 49] and it may be that these or other appraisals relating to other factors in hallucinated voices—like depth of agency or the extent to which voices seem to have individuated and characterful identities [50–52], rather than the ones measured here, are more relevant for driving distress more generally.

We also note in the longitudinal analysis that the overall severity of hallucinations reduced modestly with no convincing evidence for differences in network structure. This study did not evaluate a period of treatment change in the clinic in which it was conducted, and given that a prior study conducted in the same clinic found most patients are on stable doses of antipsychotic medication [39], we suspect that this change may reflect non-treatment-related fluctuation in the experience of auditory hallucinations—particularly given that wait lists, at least in randomised controlled trials, have been found to be modestly counter-therapeutic [53], contrary to what we found here. Previous studies have tended to show marked fluctuation over short time periods (typically over several days; [54–57]) with more stability when measured over a period of a year or more [58, 59] and it is possible that the results presented here covering an average time period of 117.2 days are the mid-point in this pattern of temporal fluctuation. However, we also note the wide range of time periods between assessments that were summarised by this mean value and more precise, time-stratified studies are needed to test this assumption more reliably. We also raise the question of whether a more radical change in structure would be apparent if networks were compared before and after treatment. Network structure has been found to predict treatment outcome in depression [36] and it is possible that this may be the case for hallucinated voices.

It is worth noting some potential limitations to the study reported here. The data are taken from routine clinical practice and may not have the same level of reliability as a specifically designed prospective study. In the longitudinal analysis, dropouts may also affect the final population of patients whose data are included in the study. Indeed, as the data were drawn from an outpatient service, this naturally selects for referrals of people who are less likely to be experiencing an acute episode of psychosis and who live in the community, which may mean the results are less representative of voices in acute episodes. Conversely, those whose difficulties remit or who decide the treatment offered by the clinic does not suit them may also be less likely to return. Although previous data shows most patients from the clinic from which the sample was drawn are on stable doses of antipsychotic medication [39], the lack of data on prescribing meant we could not estimate to what extent this affected symptom variability. Although not a limitation per se, we note that the transdiagnostic nature of this study does provide data on potential differences in the structure of hallucinated voices that are specific to certain diagnoses.

## Conclusions

We report that several appraisals and beliefs that have previously been identified as key in characterising the clinical significance of hallucinated voices in psychiatry seem to have little relation to several measures of distress and disability and seem to make little contribution to the estimated network structure. The structure of hallucinated voices changed little over time.

## Electronic supplementary material

Below is the link to the electronic supplementary material.

Supplementary file1 (DOCX 665 kb)
