# Supplementary material for: Insight-related beliefs and controllability appraisals contribute little to hallucinated voices: a transdiagnostic network analysis study
Source: Eur Arch Psychiatry Clin Neurosci. 2020 Jul 14;271(8):1525–35. doi: 10.1007/s00406-020-01166-3 (PMC8563563; doi:10.1007/s00406-020-01166-3)
Supplement: Supplementary file 1 — Supplementary file1 (DOCX 665 kb) [file 406_2020_1166_MOESM1_ESM.docx]

**Supplementary Materials**

**Study 1 Predictability Estimates**

| *PSYRATS-AH Item* | *R^2^ Predictability* |
| --- | --- |
| Amount of negative content (ANC) | 60.6% |
| Amount of distress (ADS) | 60.1% |
| Intensity of distress (IDS) | 52.6% |
| Degree of negative content (DNC) | 49.9% |
| Duration (DUR) | 31.6% |
| Disruption to life (DIS) | 26.6% |
| Frequency (FRQ) | 26.2% |
| Loudness (LDN) | 11.5% |
| Controllability (CON) | 5.7% |
| Location (LOC) | 0% |
| Belief about the origin of voices (BEL) | 0% |

**Table S1.** Predictability estimates for nodes in network of PSYRATS-AH items

**Study 1 Stability Analyses**

Stability tests test the extent to which the network and its parameters, and therefore the inferences based on them, remain robust after systematic variation and re-sampling using bootstrap sampling – as implemented in the R package *bootnet* (Epskamp et al., 2016). All analyses are run using 3000 iterations except where otherwise stated. Method descriptions modified from Bell and O’Driscoll (2018).

*Confidence intervals for edge-weights*

The edges (connections) between nodes have a weight. Using bootstrap methods, a 95% CI around the edge weights can be constructed. The graph of the bootstrap analysis is displayed in Figure S1. The red line indicates edge weight and the grey borders indicate the extent of the bootstrapped confidence intervals. Wide confidence intervals indicate low stability and confidence intervals that remain close to the value indicate high stability. Overlapping confidence intervals signify that edge weights are unlikely to significantly differ from one-another.


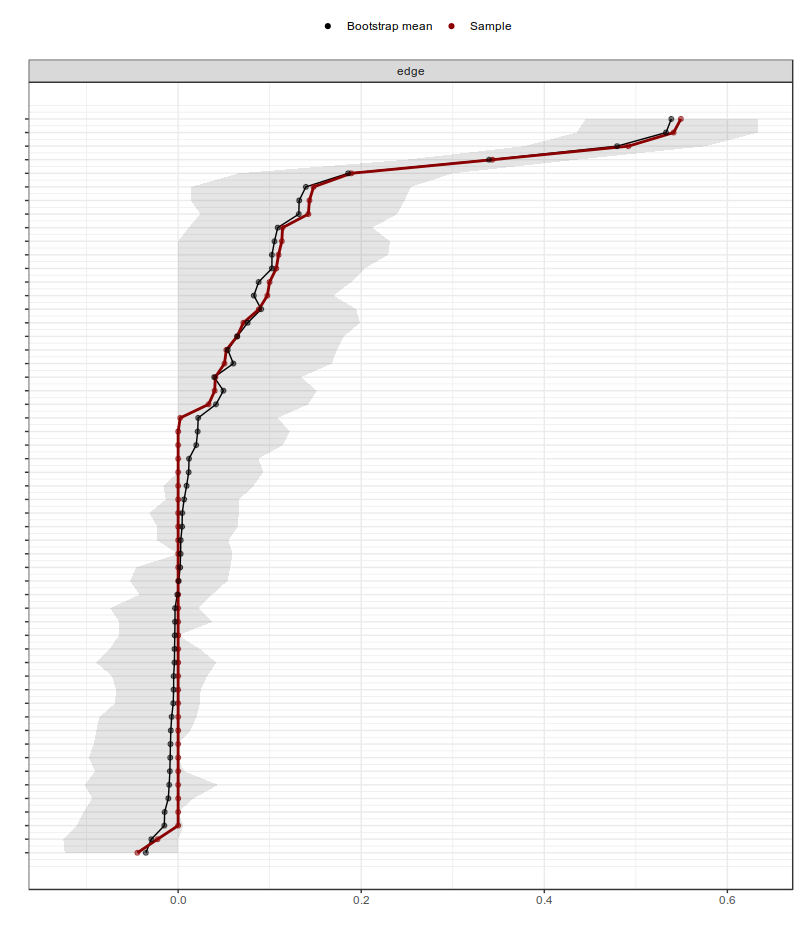


**Figure S1. Accuracy of Study 1 edge weights**

Accuracy of edges estimated with bootstrapped 95% confidence intervals. The smaller confidence intervals indicate more accurate edge estimates.

*Significant differences in node strength*

Bootstrap confidence interval tests can also estimate significant differences between the strength of any node pairing. These results are displayed in Figure S2 showing a large proportion of the node strength comparisons are significantly different (p < 0.05).


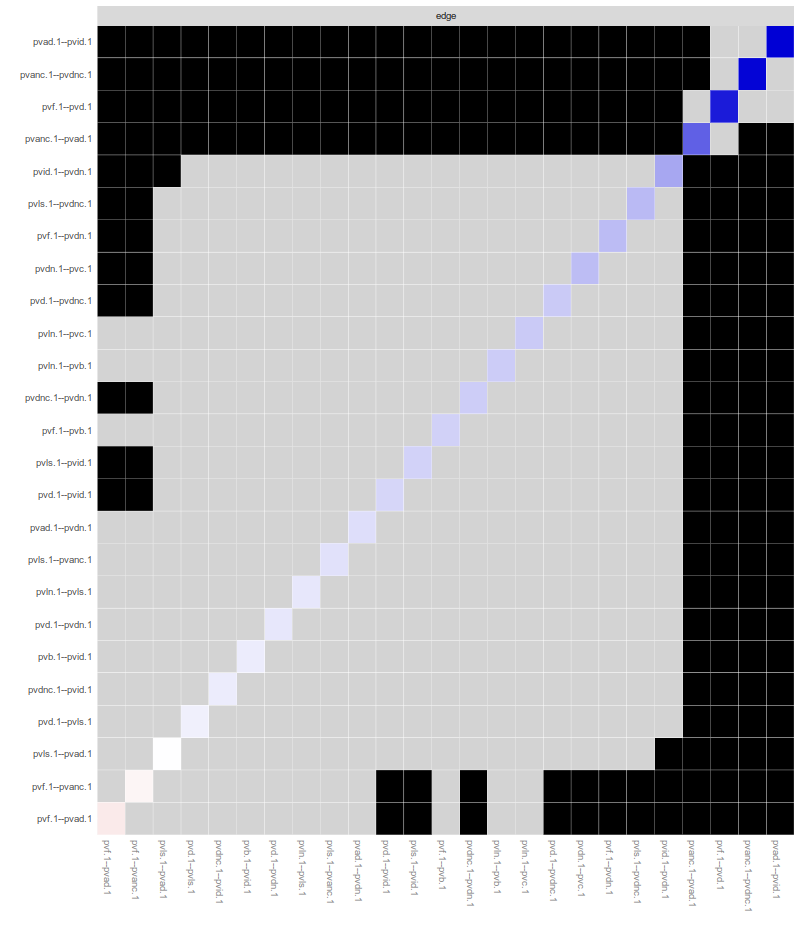


**Figure S2. Bootstrapped difference tests for Study 1 node strength**

Black represents a significant difference in node strength for each pairing, grey a non-significant difference, white the node strength value.

*Significant differences in edge-weights*

Bootstrapped difference tests can also be applied to edge-weight comparisons. Figure S3 displays significant differences between all edge-weight pairings, showing a high proportion of significant differences.


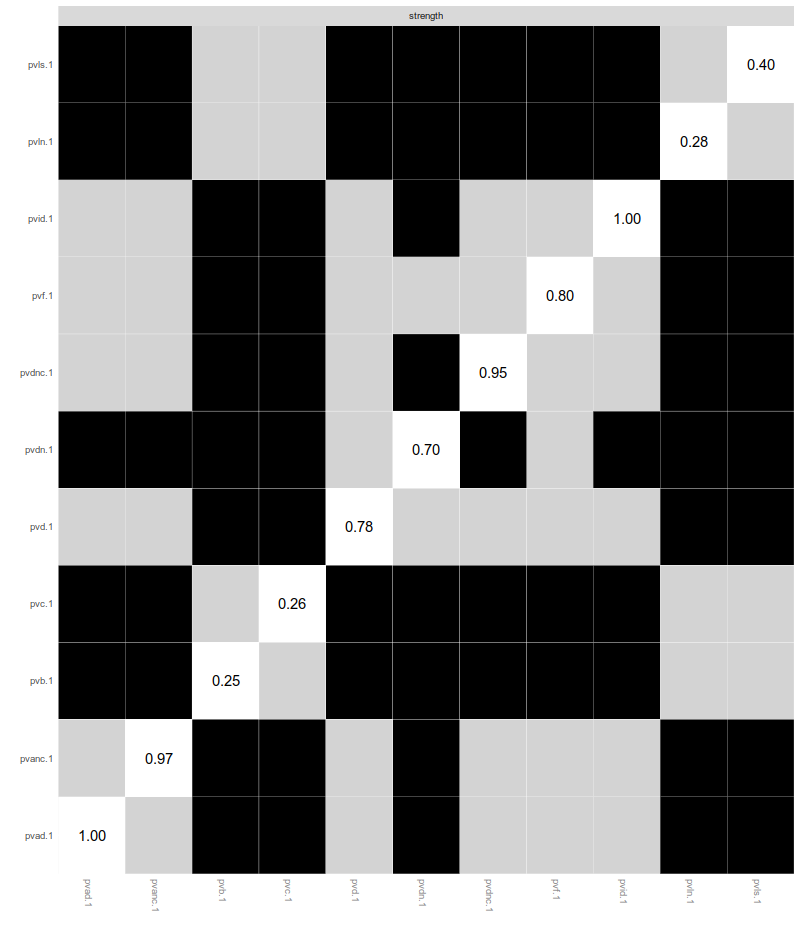


**Figure S3. Bootstrapped Study 1 edge weights difference test**

Black represents a significant difference between edge weight pairings, grey a non-significant difference.

*Stability of strength centrality metric*

The stability of the strength centrality metric can be tested by correlating the metrics obtained from the full sample with metrics obtained after systematically removing increasing numbers of cases from the analysis. Graph Figure S3 shows the stability of the strength metric during this process. Please note: As a bootstrap analysis, the stability coefficients rely on an element of random sampling.

**
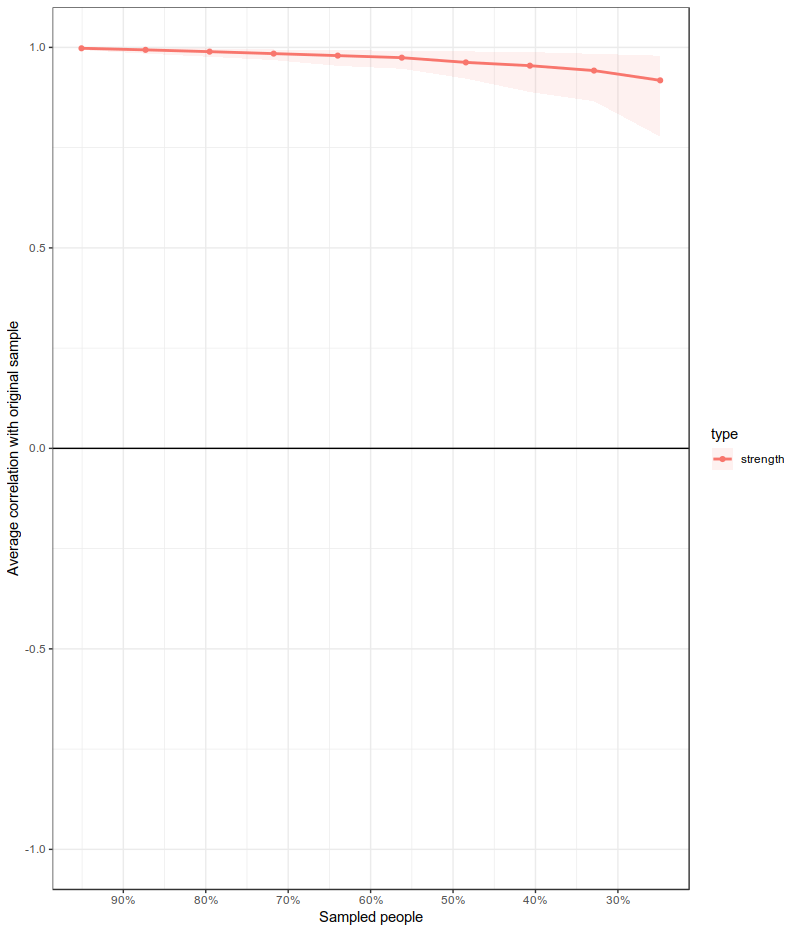
**

**Figure S4. Study 1 Strength Centrality Metric Stability**

Correlation of the strength centrality metric with metrics calculated with increasing numbers of randomly removed participants.

**
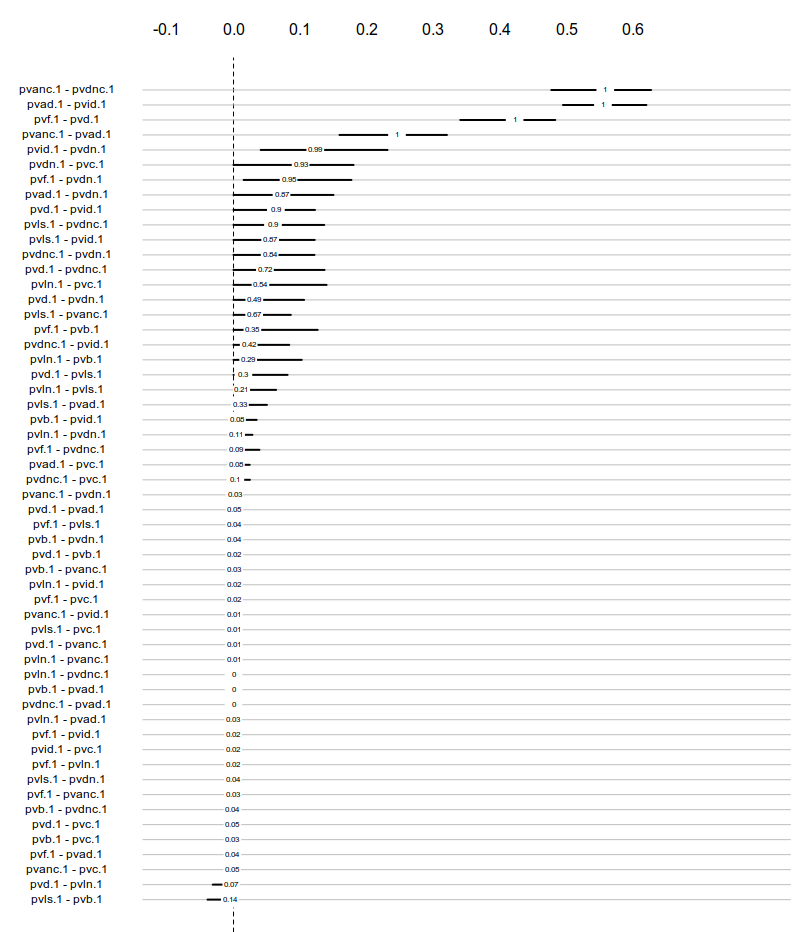
**

**Figure S5. Accuracy of Study 1 edge weights using Fried et al. (2018) method**

Accuracy of edges estimated with bootstrapped 95% confidence intervals using method for *mgm* networks developed by Fried et al (2018) (<https://osf.io/6ehrm/>). The smaller confidence intervals indicate more accurate edge estimates.

**Study 2 Stability Analyses**

Study 2 included two networks, at first assessment and second assessment.

| 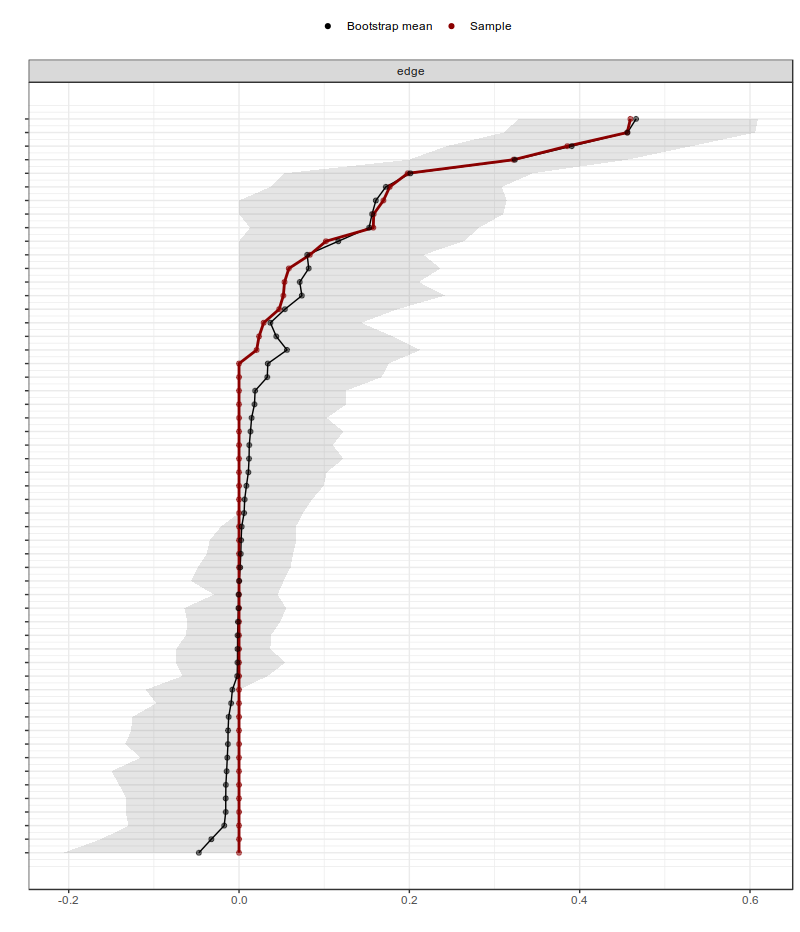  Assessment One | 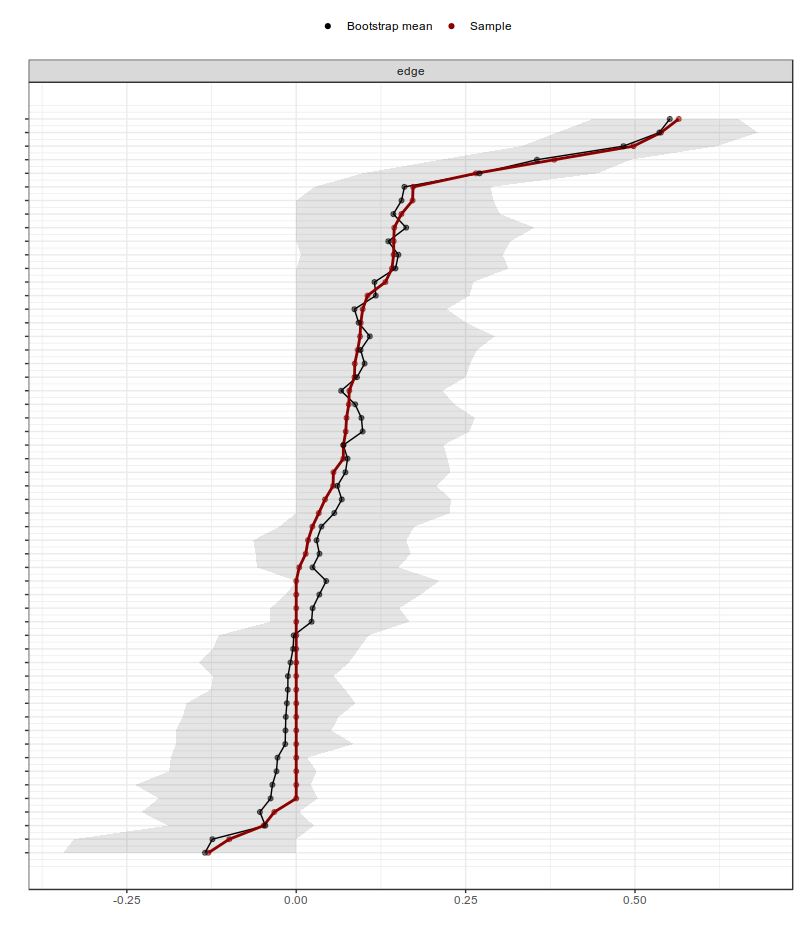  Assessment Two |
| --- | --- |

**Figure S6. Accuracy of Study 2 edge weights for assessment one and two networks**

Accuracy of edges estimated with bootstrapped 95% confidence intervals. The smaller confidence intervals indicate more accurate edge estimates.

| 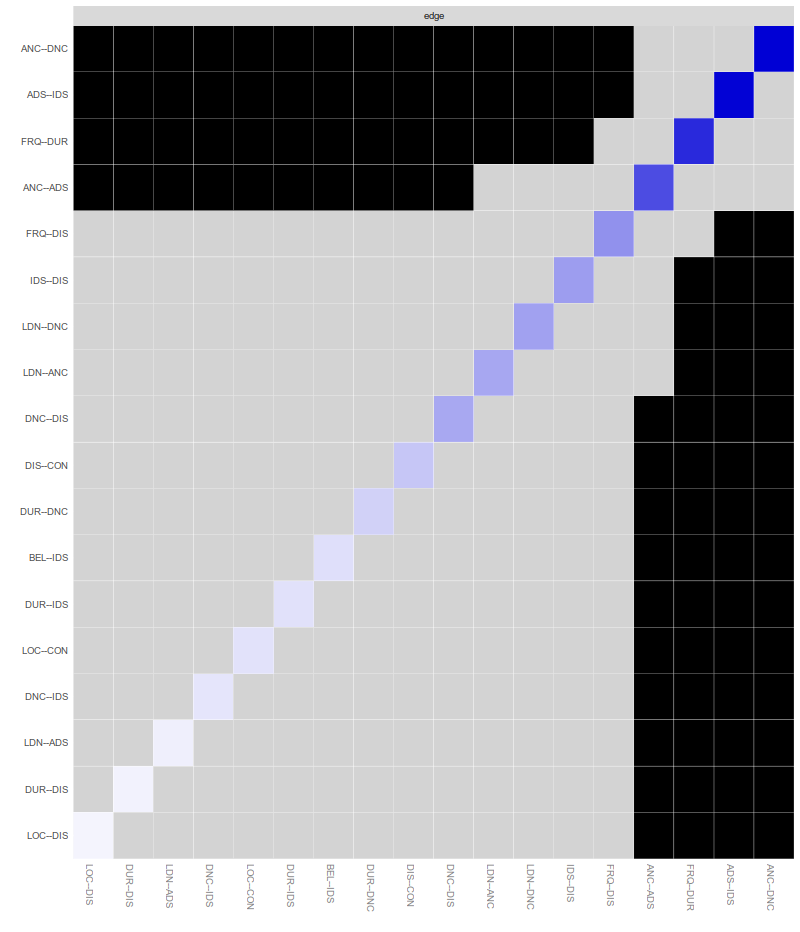  Assessment One | 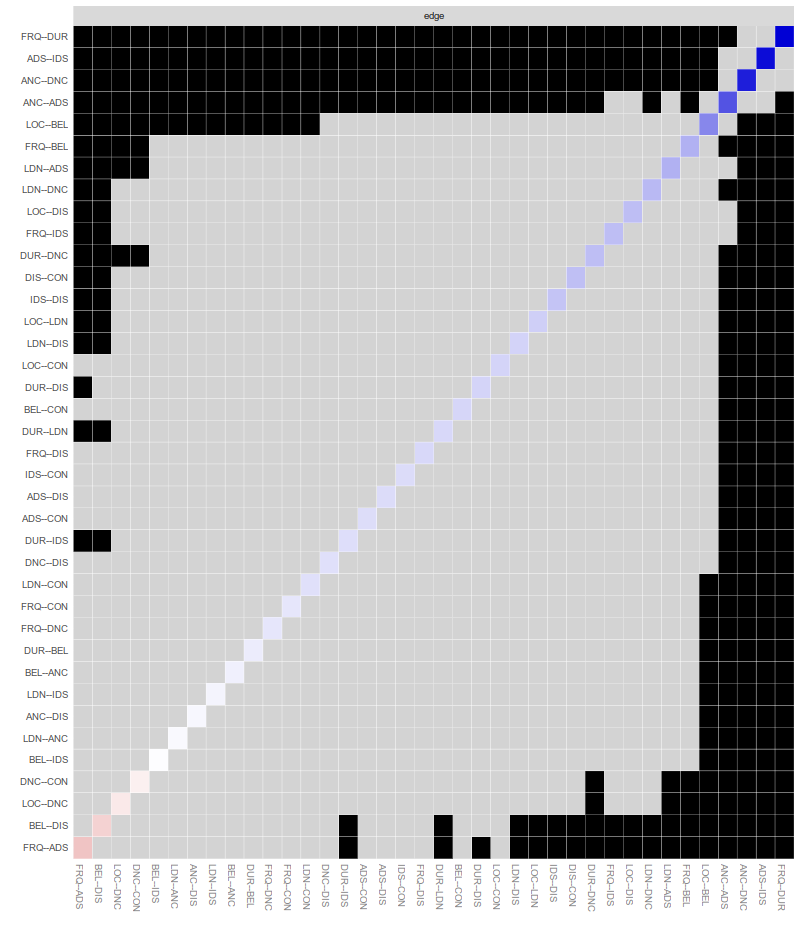  Assessment Two |
| --- | --- |

**Figure S7. Bootstrapped difference tests for Study 2 node strength for assessment one and two networks**

Black represents a significant difference in node strength for each pairing, grey a non-significant difference, white the node strength value.

| 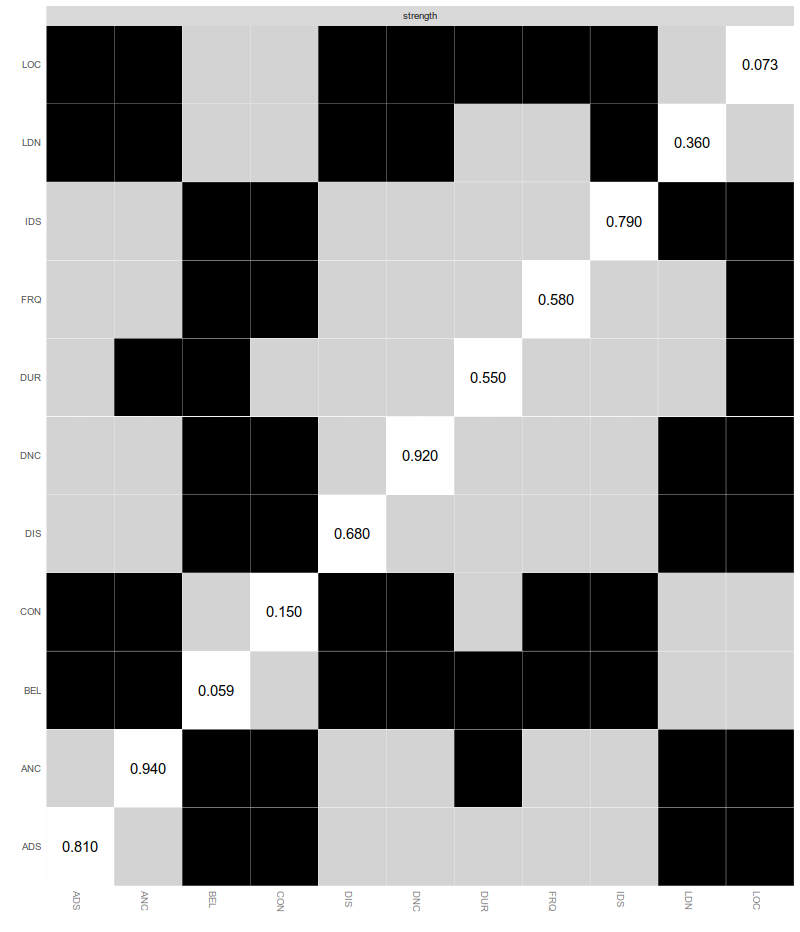  Assessment One | 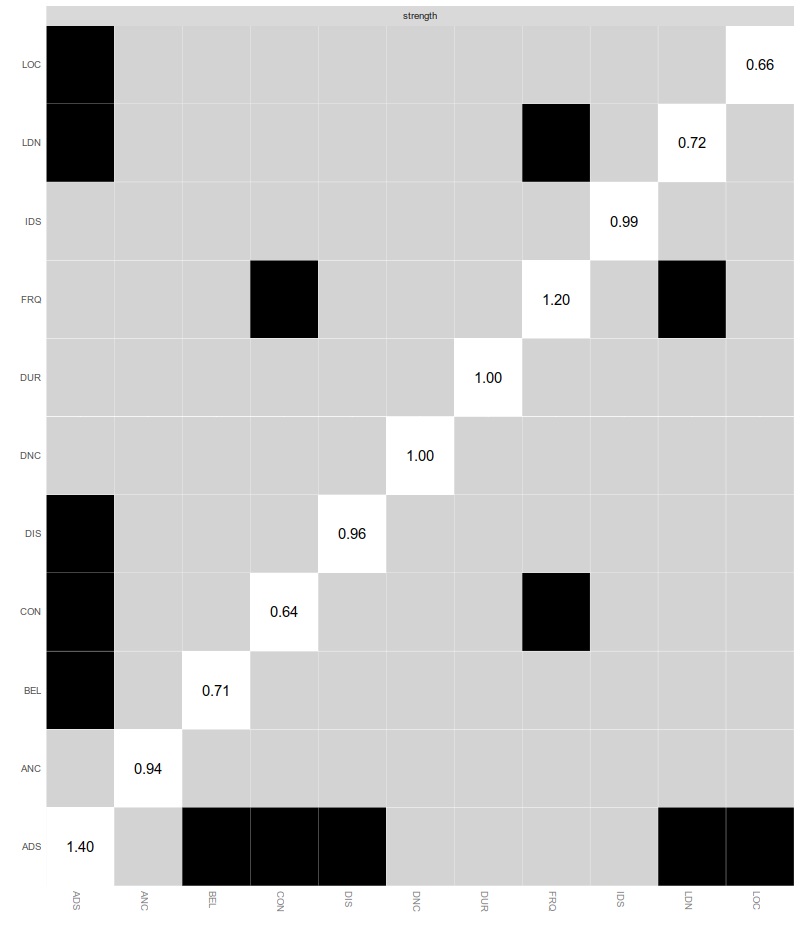  Assessment Two |
| --- | --- |

**Figure S8. Bootstrapped Study 2 edge weights difference test for assessment one and two networks**

Black represents a significant difference between edge weight pairings, grey a non-significant difference.

| 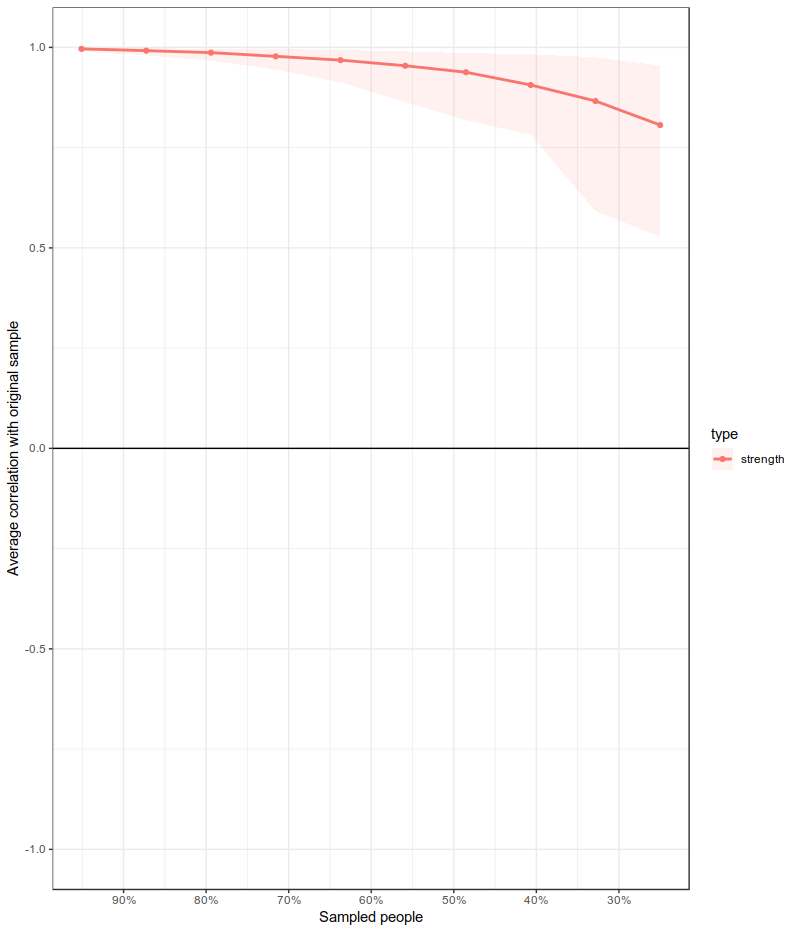  Assessment One | 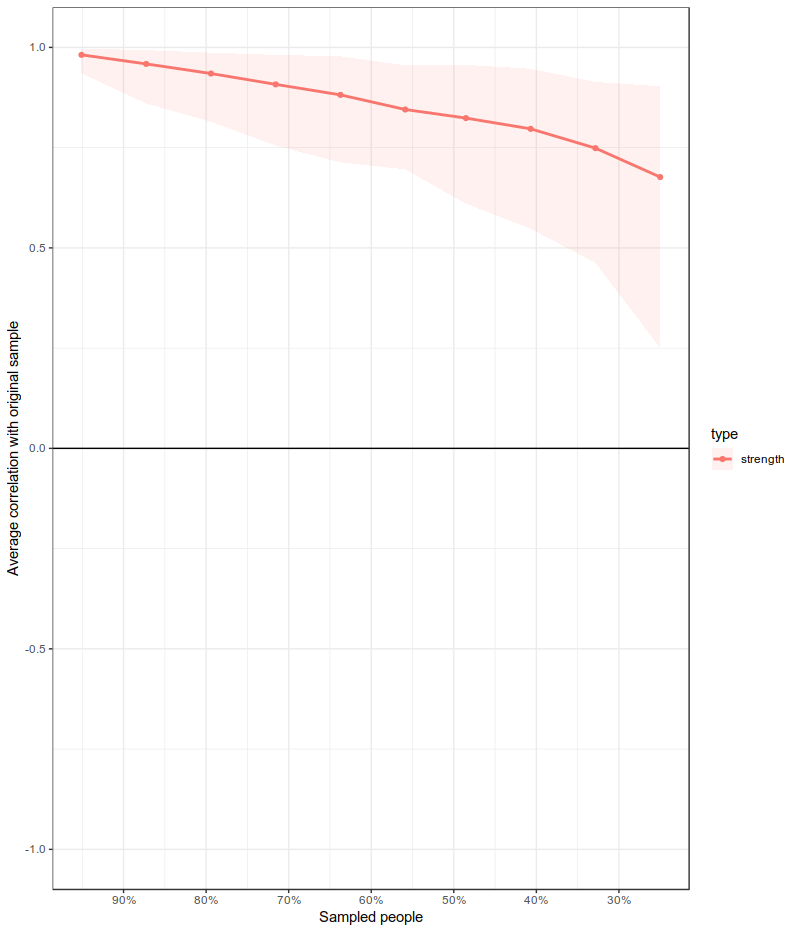  Assessment Two |
| --- | --- |

**Figure S9. Study 2 Strength Centrality Metric Stability for Assessment One and Two Networks**

Correlation of the strength centrality metric with metrics calculated with increasing numbers of randomly removed participants.

| **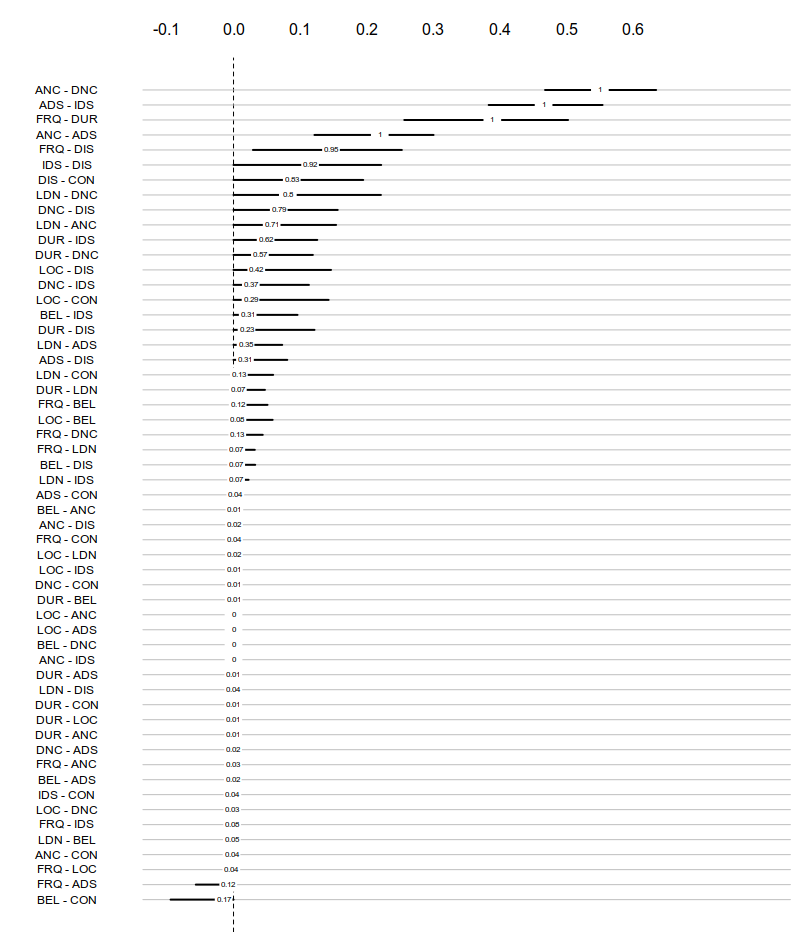**  Assessment One | **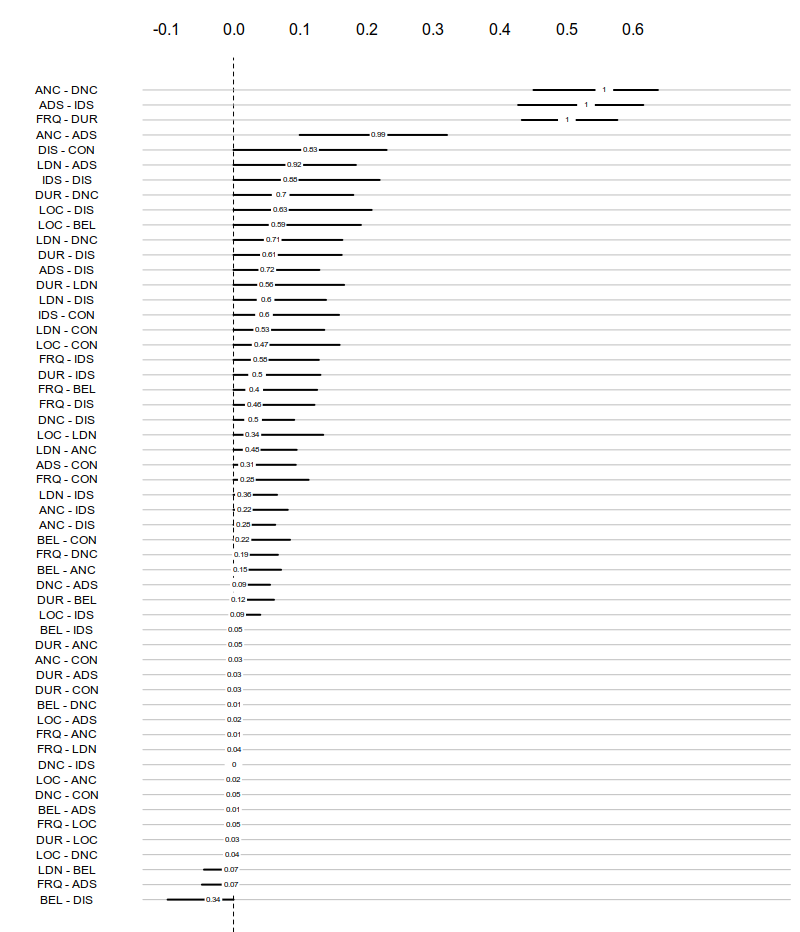**  Assessment Two |
| --- | --- |

**Figure S5. Accuracy of Study 2 edge weights using Fried et al. (2018) method**

Accuracy of edges estimated with bootstrapped 95% confidence intervals using method for *mgm* networks developed by Fried et al (2018) (<https://osf.io/6ehrm/>). The smaller confidence intervals indicate more accurate edge estimates.
